# A Case of Poisoning by Chloroform

**Published:** 1856-11

**Authors:** Junius Williams

**Affiliations:** Assistant Resident Physician, Philadelphia Hospital, Blockley


*A Case of Poisoning by Chloroform.* By Dr. JUNIUS WILLIAMS, Assistant Resident Physician, Philadelphia Hospital, Blockley.

Mary McNamee, æt. 35, Irish by birth, a woman of intemperate habits, entered the house some six weeks previously with delirium tremens, recovering from which she had been appointed an assistant in one of the wards, in which capacity she was serving at the time of the accident. On the afternoon of September 22d, she was seen intoxicated. About 9 o'clock in the evening she went to the table of a patient in ward No. 2, and took what she imagined to be a bottle of sweet spirits of nitre, but which contained in reality about f.℥jss. of chloroform, prescribed for another patient as an external application; she poured the entire quantity into a vessel she held in her hand, and diluting it with about the same amount of water swallowed it at a draught. Ten or fifteen minutes afterwards she descended to the story below, comprising the Women's Surgical Wards, and appeared quite elated, giving vent to the exuberance of her feelings by singing and dancing. Whilst turning to leave the ward, she fell completely insensible. Dr. J. K. Kane, who was kindly taking supervision over my ward during my sickness, was sent for, arrived in a few minutes, and found her respiration stertorous, the pulse feeble and rather slow, and the pupils of her eyes very much and immoveably contracted. A mustard sinapism was applied over the region of the epigastrium, dry cups were placed over the temples, and cut over the back of the neck; friction was made on the spine and heat applied to the feet. Two hours afterwards the stomach pump was used, and the contents of the stomach evacuated, after which brandy was administered through the tube. About 12 o'clock, after every remedy had been exhausted in the vain attempt to restore her, she was left with but little change in the symptoms, in which condition she remained during the remainder of the night and part of the next morning. About 7 in o'clock the morning she roused up and asked for the nurse, then bid the assistant not to disturb her as she had been sick during the night; of this fact no one had informed her, and whether she had been conscious during her protracted swoon, or whether it was a guess, I was not able to ascertain.

At 8 o'clock, the usual time of my daily visit to the wards, I first saw her; her respiration was easy but hurried, her pulse feeble and slow; she was entirely conscious, and lay upon her left side with her right arm resting upon her head; her eyes were closed, but upon being opened, the pupils were seen somewhat relaxed. She complained of a burning pain in the fauces, œsophagus and stomach. She gave me an account of her taking the chloroform, not being conscious yet of the terrible mistake she had made, but complained that it burned her very much at the time of swallowing it. I prescribed just sufficient dilute brandy to prevent her sinking, and ordered perfect rest.

My next visit was at 9 o'clock; her pulse had somewhat increased in rapidity, the other symptoms remaining much the same as during my previous visit; her breath still smelt strongly of the chloroform; she had not taken more than f.ʒij. of the brandy.

At my next visit, at 12 o'clock, I found that vomiting had set in some half an hour previously, of a dark homogeneous fluid, exceedingly offensive; she had purged also at intervals of from fifteen to twenty minutes, a fluid differing but little in color but more consistent than that thrown from the stomach, and equally offensive; her thirst was insatiable, and her pulse had gone up to one hundred beats per minute. She complained of the same burning pain in the stomach. I ordered her to be blistered over the region of the stomach, a pill of opium and acetate of lead every two hours, lime water, and small pieces of ice to be held in the mouth a short time and then swallowed, and water only to be given in small quantities at a time. During the afternoon the vomiting and purging somewhat abated, the pain still remaining as great as ever, with great aversion on her part to being moved or disturbed in any manner. I ordered Tr. Opii, forty drops, per rectum. During the night she seemed more composed.

At 8 o'clock the following morning she was throwing from the stomach a dirty greenish fluid, still offensive. I ordered a second enema of Tr. Opii, and the blister to be dressed with Morph. Sulph. gr. ss.

At 10 o'clock I was called in suddenly from an adjoining ward, and found her with the dirty green water flowing from her mouth and nostrils, her face of an intense purple, respiration and pulsation both stopped; she had died asphyxiated.

Twenty-four hours after death a port mortem examination was made.

The stomach contained about f.ʒjss. of the dirty green fluid she had been throwing up during the morning of her death; the color of the stomach was rather paler than usual, except in streaks a quarter of an inch in width, at intervals of an inch, from which I inferred that the organ had been thrown into folds by the irritation of the chloroform, and only a portion of its surface had been violently acted upon. The mucous membrane was thickened and so much softened as to be easily scraped off in shreds.

The heart contained about f.℥jss. of blood of natural color, and fluid; the commencement of the aorta was of a deeper red than usual.

The lungs presented nothing worthy of note.

The brain was healthy in color and consistence, the ventricles presenting nothing abnormal.

The veins appeared large and puffy, as though containing air; upon cutting into them the blood was perfectly fluid.

